# Aroma Characteristics and Volatile Compound Transfer in Jasmine Tea During Scenting

**DOI:** 10.3390/foods15081403

**Published:** 2026-04-17

**Authors:** Yang Yang, Ying Dong, Zhimin Song, Juanfen Zou, Xiaoqin Huang, Dezhi Mao, Chunlei He, Ling Lin

**Affiliations:** 1 Department of Tea Science, Sichuan Agricultural University, Chengdu 611130, China; 2023205046@stu.sicau.edu.cn (Y.Y.); 18783508241@163.com (Y.D.); 19960960180@163.com (Z.S.); z18313376391@163.com (J.Z.); 2 Tea Refining and Innovation Key Laboratory of Sichuan Province, Sichuan Agricultural University, Chengdu 611130, China; 3 Sichuan Kangrun Tea Industry Co., Ltd., Ya’an 625000, China; hxqin0226@163.com; 4 Miyi County Bureau of Agriculture and Rural Affairs, Panzhihua 617200, China; 18428381127@163.com

**Keywords:** jasmine tea, scenting process, aroma compounds, selective transfer

## Abstract

To reveal how the characteristic flavor of jasmine tea is generated, this study analyzed the coordinated changes in sensory properties, chemical components, and aroma migration behavior during scenting. Sensory evaluation, biochemical assays, and headspace solid-phase microextraction–gas chromatography–mass spectrometry (HS-SPME-GC-MS) integrated with orthogonal partial least squares discriminant analysis (OPLS-DA) and relative odor activity value (rOAV) filtering were applied to tea samples before and after scenting. After scenting, aroma and taste scores increased significantly, and liquor color shifted from tender green to pale yellow. Amino acids and soluble sugars increased, while astringent substances such as tea polyphenols and catechins decreased. Key floral compounds, including cis-3-hexenyl benzoate and methyl anthranilate, were transferred from jasmine flowers to the tea base and enriched, likely contributing to the typical aroma profile. The retention rate of aroma in spent flowers was positively correlated with hydrophobicity (logP, r > 0.46, *p* < 0.01) and negatively with polarity (TPSA, r > −0.42, *p* < 0.05), suggesting regulation by hydrophobic partitioning. In contrast, aroma transfer to the tea base showed no simple correlation with any single physicochemical parameter, suggesting multi-factor regulation. This study provides insights into the scenting process and offers a theoretical reference for quality control in jasmine tea production.

## 1. Introduction

Jasmine tea is a kind of reprocessed tea made by blending tea base with fresh jasmine flowers through the scenting process. With its intense and elegant aroma and mellow taste, it is deeply favored by consumers. In recent years, the jasmine tea industry in China has experienced sustained and steady development. According to statistics from the China Tea Marketing Association (accessible at https://www.ctma.com.cn/, accessed on 8 December 2025), the total domestic sales volume of jasmine tea in China reached 127,600 tons in 2024, with a total domestic sales value of 25.259 billion RMB. Both the export volume and value also grew synchronously, indicating robust market demand and promising industrial prospects.

Aroma plays a crucial role in determining the overall quality of jasmine tea, with “scenting” being the key process that forms its unique flavor [1]. The essence of this process lies in the synergistic effect of physical and chemical adsorption by the tea base of the dynamically released volatile aroma compounds from fresh jasmine flowers. The tea base for jasmine tea is primarily green tea, whose porous matrix exhibits a strong adsorption affinity for aroma substances. This adsorption process constitutes the physicochemical foundation for the characteristic aroma profile of jasmine tea [2,3]. To achieve a rich and persistent aroma, high-quality jasmine tea often undergoes multiple rounds of scenting. This not only prolongs the production cycle but also entails the consumption of large quantities of fresh jasmine flowers, with the spent flowers rarely being reused, leading to high production costs [4,5,6]. This situation underscores a key industrial and scientific issue: the aroma utilization efficiency of jasmine flowers needs to be clarified, yet a systematic quantitative understanding is currently lacking.

Current research has largely focused on areas such as the identification of characteristic aroma compounds in jasmine tea [7,8,9,10]. Recent studies employing GC-O have further advanced this field by pinpointing key contributors to jasmine tea aroma [11], while comprehensive volatilomics analyses have led to the construction of detailed aroma wheels [12]. Other research has addressed the optimization of scenting process parameters [7,13] and the compatibility of different tea bases with jasmine varieties [14,15]. However, a key question remains systematically unanswered in practical production: during dynamic jasmine scenting, the proportion of floral volatiles released by jasmine flowers that are effectively adsorbed by the tea base and contribute to the final tea aroma, as well as the proportion remaining unutilized in spent flowers, has yet to be clearly quantified. Quantitative analysis of this process of aroma transfer and allocation serves as a direct basis for understanding adsorption efficiency, evaluating raw material utilization, and optimizing the process. Nevertheless, systematic quantitative analysis and mechanistic exploration in this area remain insufficient.

This study aims to quantitatively elucidate the dual migration patterns of aroma compounds during the scenting process: their transfer from jasmine flowers to the tea base, and their residual loss in the spent flowers. To achieve this, we systematically analyzed the quality evolution from the tea base to jasmine tea, together with the changes in volatile components across the complete transfer pathway (jasmine flower → tea base → jasmine tea → spent flower). Particular attention was paid to the selective adsorption behavior of the tea base toward aroma compounds with different physicochemical properties. These results are anticipated to offer theoretical references and experimental data support for clarifying the mechanism underlying the characteristic flavor of jasmine tea, and to promote more efficient utilization of aromatic components from fresh flowers.

## 2. Materials and Methods

### 2.1. Collection and Preparation of Tea Samples

#### 2.1.1. Experimental Materials

Jasmine flowers of the double-petal variety (*Jasminum sambac* L.) Ait.) were harvested on 18 September 2024, from a local plantation in Jiajiang County, Leshan City, Sichuan Province, China. This variety is autochthonous to the region and has been cultivated there for decades. The fresh flowers were delivered to the laboratory on the evening of the picking day. After being conditioned until approximately 80% of the flowers had reached a half-bloom state, they were sealed and stored in a −20 °C freezer for subsequent use.

Tea material: The tea base was provided by Sichuan Kangrun Tea Co., Ltd. (Ya’an, Sichuan, China). It was a pan-fired green tea produced from one bud with two leaves sourced from Ya’an City, with an initial moisture content of 4%. Processing equipment: A drum roaster manufactured by Fujian Jiayou Tea Machinery Intelligent Technology Co., Ltd. (Jiayou Tea Machinery, Anxi, Fujian, China) was used to process the tea base.

All samples were obtained from a single production batch to ensure consistency.

#### 2.1.2. Scenting Process

**Figure 1:**
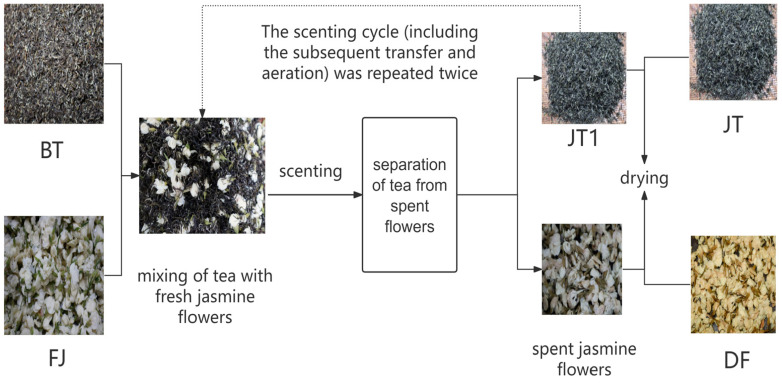
Schematic diagram of the jasmine tea scenting process flow. BT, tea base (before scenting); FJ, jasmine flowers; DF, dried jasmine flowers; JT, jasmine tea.

The scenting procedure of jasmine tea was performed mainly with reference to the national standard GB/T 34779-2017, Technical Specification for Jasmine Tea Processing [16], and further optimized based on results from preliminary experiments. Scenting was carried out in a constant-temperature and -humidity incubator, with the environmental conditions strictly controlled at a temperature of 38 °C and a relative humidity of 80%. A three-round scenting process was adopted in this experiment. The key process parameters for each scenting round were as follows: the flower-to-tea ratios (by mass) were 0.9, 0.6, and 0.5, respectively, and the scenting durations were 10.5 h, 10.5 h, and 8 h, respectively. Drying was not performed after the first and second scenting rounds; instead, a “continuous scenting” approach was employed, where the tea was directly transferred to the subsequent scenting round. After the third scenting round, tea leaves were separated from the spent jasmine flowers. The spent flowers were dried at 60 °C to obtain dried jasmine flowers (denoted as DF), while the tea leaves were baked at 110 °C until the moisture content fell below 7% to produce the jasmine tea product (denoted as JT). The tea base, jasmine flowers, dried jasmine flowers, and the jasmine tea were labeled as BT, FJ, DF, and JT, respectively. The specific process flow is illustrated in Figure 1.

### 2.2. Sensory Evaluation Method for Jasmine Tea

Five trained panelists (two men and three women) conducted sensory evaluation of the tea base and jasmine tea samples. All procedures followed the guidelines of GB/T 23776-2018, the national standard for tea sensory analysis in China [17]. All panelists received standardized training to ensure scoring consistency.

The GB/T 23776-2018 protocol includes five attributes: appearance (20%), liquor color (5%), aroma (35%), taste (30%), and infused leaf (10%). Since the present work focuses on quality variation before and after scenting, and the scenting process has negligible influence on appearance and infused leaf, these two attributes were excluded from evaluation. Their combined weight (30%) was reallocated to the remaining attributes, with priority given to aroma as the most critical quality factor for jasmine tea. The final weight coefficients were assigned as follows: liquor color 10%, aroma 50%, and taste 40%.

A standard infusion procedure was followed: 3.0 g of the representative tea sample was infused with boiling water in a tasting cup for 3 min, and the liquor color, aroma, and taste were evaluated. Subsequently, a second infusion was performed on the same leaf residue for 5 min, and the liquor color, aroma, and taste were evaluated. Panelists provided a single comprehensive score for each attribute based on the overall performance across both infusions.

Weighted scores were calculated by multiplying each attribute’s comprehensive score by its corresponding weight coefficient. The Total sensory score for each sample was then derived by aggregating these three weighted values:

(1) $$Total\;sensory\;Score=\left(Liquor\;color\times 10\%\right)+\left(Aroma\times 50\%\right)+\left(Taste\times 40\%\right).$$

Final scores were designated as BT (tea base) and JT (jasmine tea).

### 2.3. Determination of Main Biochemical Components

To elucidate the influence of the scenting process on the main biochemical components of tea, the contents were determined in both the tea base and the jasmine tea. The samples were designated as tea base (BT) and jasmine tea (JT). All results are expressed as mass fractions on a dry weight basis. For intuitive comparison in figures, the contents of all components were uniformly converted to percentages (%), i.e., grams per 100 g dry weight (g/100 g). For components originally quantified in milligrams per gram (mg/g), such as catechins and total flavonoids, the values were divided by 10 to convert to percentages.

The specific determination items and methods were as follows: Moisture: Determined according to GB 5009.3-2016 [18] (direct drying method). Water extract: Content determination was performed following the standard procedure specified in GB/T 8305-2013 [19]. Tea polyphenols were measured in accordance with GB/T 8313-2018 [20] using gallic acid as the reference standard. Caffeine: Determined according to GB/T 8312-2013 [21] (ultraviolet spectrophotometry), with caffeine as the standard. Total free amino acids: Determined according to GB/T 8314-2013 [22], with theanine as the standard. Soluble sugars [23]: Determined by the anthrone-sulfuric acid colorimetric method, with glucose as the standard. Total catechins [23]: Determined by the vanillin-hydrochloric acid colorimetric method. Total flavonoids [24]: Determined by the aluminum chloride colorimetric method. All standards (gallic acid, caffeine, theanine, and glucose) were acquired from Sinopharm Chemical Reagent Co., Ltd. (Shanghai, China), and their purity was no less than 99%. All colorimetric and spectrophotometric analyses were performed using a UV-Vis spectrophotometer (Model UV754N, Shanghai Instrument Analytical Instrument Co., Ltd., Shanghai, China), following the corresponding national standards cited above. Other reagents, including Folin–Ciocalteu reagent, anthrone, vanillin, and aluminum chloride, were of analytical grade and acquired from mainstream chemical suppliers. Among them, Folin–Ciocalteu reagent was provided by Beijing Solarbio Science & Technology Co., Ltd. (Beijing, China), whereas other reagents were obtained from Sinopharm Chemical Reagent Co., Ltd. (Shanghai, China) and Chengdu Kelong Chemical Co., Ltd. (Chengdu, China).

### 2.4. Analysis of Volatile Components

#### 2.4.1. Sample Pretreatment and SPME Extraction

Samples preserved at −80 °C were ground into a homogeneous powder under liquid nitrogen. A 500 mg portion of the ground sample was transferred into a 20 mL headspace vial (Agilent, Palo Alto, CA, USA), to which 2 mL of saturated NaCl solution (analytical grade, Sinopharm Chemical Reagent Co., Ltd., Shanghai, China) and 20 μL of internal standard (10 μg/mL) were successively added. The vial was then sealed tightly using a TFE-silicone septum cap (Agilent).

For automated headspace solid-phase microextraction (HS-SPME), the vial was subjected to equilibration at 60 °C for 5 min. After preconditioning a 120 μm DVB/C-WR/PDMS SPME Arrow fiber at 250 °C for 5 min, the fiber was placed in the headspace at 60 °C for 15 min to trap volatile compounds. It was then introduced into the Agilent 8890 GC inlet and thermally desorbed at 250 °C for 5 min for subsequent analysis [14].

#### 2.4.2. GC-MS Analysis Conditions

Analysis was carried out using an Agilent 8890-7000D GC-MS system (Santa Clara, CA, USA) coupled with a DB-5MS capillary column (30 m × 0.25 mm × 0.25 μm, Agilent J&W Scientific, Folsom, CA, USA). High-purity helium (≥99.999%) was employed as the carrier gas at a constant flow rate of 1.2 mL/min. The injector port was maintained at 250 °C under splitless injection mode, with a solvent delay time of 3.5 min.

The column oven temperature was programmed as follows: held at 40 °C for 3.5 min, ramped to 100 °C at 10 °C/min, further raised to 180 °C at 7 °C/min, and finally increased to 280 °C at 25 °C/min with a 5 min hold.

Mass spectrometry was performed in electron ionization (EI) mode at 70 eV. The ion source temperature was set to 230 °C, the quadrupole temperature to 150 °C, and the interface transfer line to 280 °C. Data collection was conducted in selected ion monitoring (SIM) mode [25].

### 2.5. Statistical Analysis Methods

The scenting experiments were conducted using a single production batch, with all samples derived from the same batch of tea base and the same batch of fresh jasmine flowers. Three technical replicates (i.e., three independent extractions and analyses per sample) were performed for each sample type, and all data are presented as mean ± standard deviation. Analysis of variance (ANOVA) was conducted using SPSS software (version 27.0, IBM, Armonk, NY, USA). Principal component analysis (PCA) plots, bar charts, and stacked plots were generated using Origin Pro software (version 2025b, Origin Lab, Northampton, MA, USA), while heat maps were generated using the MetWare cloud platform (https://cloud.metware.cn), accessed on 11 April 2026. Orthogonal partial least squares discriminant analysis (OPLS-DA) was conducted using SIMCA v14.1 (Umetrics, Umeå, Sweden) to compare metabolite profiles between sample groups. Model validation was conducted using six-fold cross-validation, with the predictive ability assessed by the Q^2^ value. Additionally, 200 permutation tests were performed to evaluate the risk of overfitting. Variable importance in projection (VIP) values, along with corresponding *p*-values, were calculated to identify differential metabolites. For correlations between two variables, Spearman’s rank correlation analysis was employed.

#### 2.5.1. Determination of Metabolite Composition and Content

Chromatographic peak integration and correction were performed using Agilent Mass Hunter Quantitative Analysis software (version B.08.00, Agilent Technologies, Santa Clara, CA, USA).

Qualitative Analysis: Volatile compounds were identified using a comprehensive approach. An in-house database was constructed using authentic standards, the NIST 2020 library, retention indices (RIs), and literature data [26]. For each compound, one quantitative ion and 2–3 qualitative ions were selected for SIM analysis.

Identification was performed using Agilent Mass Hunter Qualitative Analysis software with a minimum matching factor of 70. Volatile compounds were initially identified by matching their mass spectra with the NIST 2020 database, and their identities were further verified by comparing the experimentally determined retention indices (RIs) with corresponding literature data. RI values were computed with a homologous series of n-alkanes (C7–C40, Sigma-Aldrich, Merck KGaA, Darmstadt, Germany) as external references, following the conventional linear temperature-programmed RI method. Compounds meeting the retention time and characteristic fragment criteria were considered positively identified.

Quantitative Analysis: Quantification was performed using an internal standard method with 3-hexanone-2,2,4,4-d_4_ as the internal standard (purity ≥ 99%; C/D/N Isotopes Inc., Pointe-Claire, QC, Canada; product code D-8095). We calculated the relative content (*Xᵢ*, μg/g) according to Equation (2):

(2) $$Xi=\frac{Vs\times Cs}{M}\times \frac{Ii}{Is}\times 10^{-3},$$

where *Vs* is the volume of the added internal standard (μL), *Cs* is the concentration of the internal standard solution (μg/mL), *M* is the mass of the test sample (g), *Ii* is the chromatographic peak area of compound i, and *Is* is the chromatographic peak area of the internal standard.

#### 2.5.2. Calculation of Evaluation Parameters for Key Aroma Compounds

To evaluate the contribution of each volatile to the overall sample aroma, the relative odor activity value (rOAV) combines the compound’s concentration with its sensory threshold [27]. It integrates both the concentration and the odor threshold of a compound to evaluate its contribution to the overall aroma profile of a sample.

The rOAV for any given compound i was derived using Equation (3):

(3) $$rOAVi=\frac{Ci}{Ti},$$

In this equation, *Ci* represents the relative concentration of compound i in the sample (μg/g), and *Ti* stands for its aroma threshold in water or a comparable medium (μg/g). The threshold values employed in this work were mainly obtained from previously published reports.

#### 2.5.3. Evaluation of Material Transfer and Retention During the Scenting Process

To assess how efficiently aroma compounds move from jasmine flowers into tea leaves over the course of scenting, the Transfer Rate (TR) was defined and calculated using Equation (4):

(4) $$TR=\frac{C_{JT}-C_{BT}}{C_{FJ}}\times 100\%,$$

where *C*_JT_ represents the relative content of the target compound in jasmine tea (μg/g), *C*_BT_ is the relative content of the compound in the tea base (μg/g), and *C*_FJ_ denotes its relative content in jasmine flowers (μg/g).

To assess the residual status of the original aroma components from jasmine flowers in the dried jasmine flowers (DF) after scenting, the Retention Rate (RR) was defined and calculated according to Formula (5):

(5) $$RR=\frac{C_{DF}}{C_{FJ}}\times 100\%,$$

where *C*_DF_ is the relative content of the compound in dried jasmine flowers (μg/g), and *C*_FJ_ is the relative content of the compound in jasmine flowers (μg/g).

#### 2.5.4. Acquisition of Physicochemical Property Parameters and Correlation Analysis

Physicochemical parameters of volatile compounds, including boiling point (BP), logP (octanol-water partition coefficient), molecular weight (MW), TPSA (topological polar surface area), HBD (hydrogen bond donor count), HBA (hydrogen bond acceptor count), and ACD/KOC (organic carbon-water partition coefficient), were retrieved from public chemical databases. The primary query platforms were ChemSpider (https://www.chemspider.com/), accessed on 1 November 2025 and PubChem (https://pubchem.ncbi.nlm.nih.gov/), accessed on 1 November 2025. Spearman’s rank correlation analysis was subsequently performed based on the acquired parameters.

## 3. Results

### 3.1. Effect of Scenting on the Sensory Quality and Main Biochemical Components of Jasmine Tea

**Figure 2:**
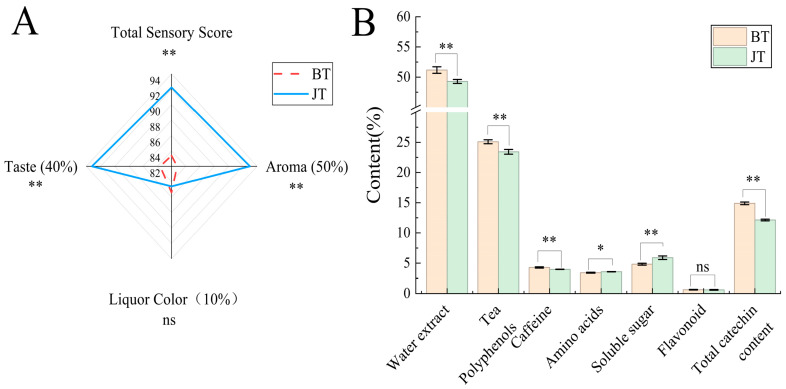
Sensory evaluation and biochemical components of jasmine tea before and after scenting. (**A**) Radar chart of sensory attributes; (**B**) Bar chart showing changes in the content of main biochemical components (* *p* < 0.05, ** *p* < 0.01, ns *p* > 0.05). BT, tea base (before scenting); JT, jasmine tea.

The quality of jasmine tea primarily depends on its fresh and rich aroma [28], followed by its mellow and sweet taste, while the liquor color mainly affects the visual experience during consumption. The scenting process systematically alters the sensory quality of the tea. As shown in Figure 2A, the aroma and taste scores exhibited a significant increasing trend after scenting, whereas the liquor color score showed a slight decrease (a reduction of 0.94%), which did not reach a statistically significant level. Specifically, the aroma score increased by 12.32%, the taste score by 11.48%, and the total score by 10.63%, indicating that aroma is a key factor driving the quality improvement of jasmine tea. This change may be related to the alternating effect of “adsorption-drying” during the scenting process: during the drying step, some volatile aroma compounds are lost, while others become more firmly adsorbed within the tea leaves; subsequent scenting rounds further promote the integration and redistribution of floral compounds with the tea base. The taste score exhibited a trend of increase similar to that of the aroma score, reflecting that under the hot and humid conditions of scenting, the taste characteristics of the tea base gradually shift from the strong astringency of green tea to the characteristic freshness, mellowness, and harmony of jasmine tea.

Concurrently, the scenting process also induced significant transformations in the main biochemical components of the tea (Figure 2B). Components such as water extract, tea polyphenols, and free amino acids constitute the key material basis that determines the flavor quality of tea [29,30]. As shown in Figure 2B, after scenting, each component exhibited regular patterns of change: the contents of tea polyphenols, total catechins, and caffeine decreased significantly (with reductions ranging from 6.62% to 18.47%), the total free amino acids content increased by 4.99%, and the soluble sugar content increased significantly by 22.20%. Compared with the pre-scenting tea base (BT), various indicators of jasmine tea (JT) underwent significant changes (*p* < 0.05): the contents of water extract, tea polyphenols, caffeine, and total catechins decreased to 49.29%, 23.43%, 3.97%, and 12.14%, respectively, while the contents of free amino acids and soluble sugars increased to 3.58% and 5.89%, respectively; the change in flavonoid content was not significant (*p* > 0.05).

The aforementioned changes in biochemical components provide the material basis for the transformation of sensory quality. The increase in total free amino acids and soluble sugars may synergistically contribute to the taste characteristics of freshness, mellowness, sweetness, and briskness, whereas the significant reduction in astringent substances such as tea polyphenols and catechins contributes to a milder and smoother tea liquor [31,32]. After scenting, the liquor color score decreased slightly, with the tea liquor gradually shifting from the tender green and bright of the tea base to pale yellow and bright. This visually reflects the transformation of the tea’s intrinsic components under hot and humid conditions. During this process, the tea leaves continuously adsorb and integrate volatile aroma compounds from jasmine flowers. This is likely the result of a combination of physical adsorption and chemical interactions. Further investigation is needed to elucidate the underlying mechanisms.

In summary, the scenting process not only significantly enhances the aroma of jasmine tea but also optimizes its taste by influencing the internal chemical components, collectively shaping the typical quality style of jasmine tea characterized by a “fresh and rich” aroma and a mellow, sweet, and brisk taste.

### 3.2. Analysis of Aroma Compounds in Jasmine Tea During the Scenting Process

#### 3.2.1. Composition and Overall Profile Analysis of Aroma Compounds

Volatile components in the tea base (BT), jasmine flowers (FJ), jasmine tea (JT), and dried jasmine flowers (DFs) were analyzed by HS-SPME-GC-MS. A total of 188 volatile compounds were identified across the four samples and classified into 12 chemical classes. The most abundant classes were terpenes (61 compounds) and esters (53 compounds), followed by ketones (25 compounds) and aldehydes (24 compounds). Other classes included alcohols (11 compounds) and heterocyclic compounds (6 compounds). Detailed identification data and relative contents are listed in Supplementary Table S1.

**Figure 3:**
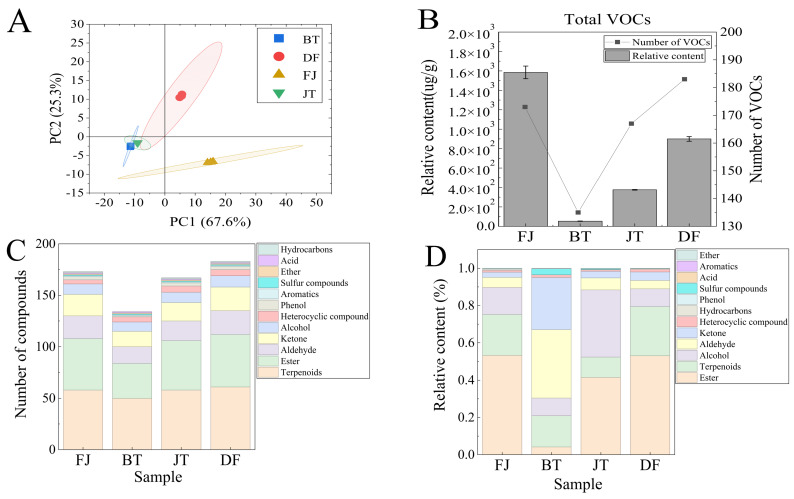
Distribution of aroma compounds in tea base (BT), jasmine flowers (FJ), jasmine tea (JT), and dried jasmine flowers (DF) during the scenting process. (**A**) Principal component analysis (PCA) score plot for volatiles across samples; (**B**) Total content and count of volatile compounds per sample; (**C**) Counts of volatiles categorized by chemical class for each sample; (**D**) Relative abundance of each chemical class among volatiles per sample.

Principal component analysis (PCA) was performed to characterize differences in aroma composition among samples, with the results shown in Figure 3A. The PCA score plot revealed obvious intergroup separation and intragroup clustering. PC1 (67.6%) and PC2 (25.3%) together explained 92.9% of the total variance, indicating that the model sufficiently reflected sample differences. FJ and DF were clearly separated along the PC2 axis, implying that the scenting and drying processes induced substantial changes in the volatile composition of DF. JT and DF exhibited a large overlap in their confidence ellipses, suggesting a similar aroma profile. Meanwhile, BT and JT showed partial overlap, indicating a certain correlation in their overall volatile composition.

As shown in Figure 3B, FJ displayed the highest total content of volatile compounds, while DF possessed the greatest number of volatile components. In contrast, BT exhibited both the lowest content and the fewest compounds. Following the scenting process, the content and number of volatiles in JT increased markedly, whereas DF still retained a considerable amount of volatile components.

Among the identified volatile compounds, the quantitative distribution of various substances differed across samples, but terpenes and esters were quantitatively dominant in all cases (Figure 3C), consistent with previous studies [7,8]. The major aroma compounds found in our jasmine tea preparation showed good agreement with previously published data [10,28,33]. However, indole was not detected, potentially due to differences in variety, physiological state, or analytical methods [34,35].

Analysis of the relative content of different classes of compounds (Figure 3D) revealed characteristic distribution patterns among the samples. Both FJ and DF exhibited esters as the predominant volatile component (approximately 53% each), followed by terpenes (FJ: 22%; DF: 26%) and alcohols (FJ: 15%; DF: 9%), indicating that jasmine flowers retain substantial flavor compounds after releasing their aroma. BT, in contrast, was dominated by aldehydes (37%), ketones (28%), and terpenes (17%). After scenting, the volatile composition of JT underwent a marked shift: esters (42%) and alcohols (36%) became the most predominant components, followed by terpenes (11%), while the aldehyde content showed a substantial reduction. This trend indicates that scenting essentially involves the transfer and enrichment of characteristic aroma compounds like esters and alcohols from jasmine flowers into tea leaves, accompanied by a dynamic decrease in the relative proportion of volatile substances such as aldehydes inherent to the tea leaves [36].

#### 3.2.2. Global Screening of Aroma-Active Compounds Based on rOAV

The above analysis revealed the distribution patterns of volatile compounds based on relative content. However, the content of a compound does not directly equate to its contribution to the overall aroma, as compounds with high relative contents may not necessarily be aroma-active, whereas those with low contents but extremely low odor thresholds could play pivotal roles. Therefore, to further identify the key aroma-active compounds during the scenting process, a global screening based on relative odor activity value (rOAV) was performed.

Among the 188 volatile compounds identified, 142 had reported odor thresholds in water and were distributed across 11 chemical classes (Supplementary Table S2). The hydrocarbon class contained only one compound, (Z)-2,3-dimethyl-3-heptene, for which no threshold was reported; it was therefore excluded from the rOAV analysis. The representative compounds with the highest rOAV in each chemical class, together with the top five terpenes in JT, are summarized in Table 1 and Table 2.

The above results indicate that ketones exhibited the highest rOAV in jasmine tea, with dihydro-2-methyl-3(2H)-furanone showing exceptionally high activity in JT (570,251). Notably, this sweet aroma compound exhibited the highest rOAV in BT, suggesting that it primarily originates from the tea base itself. Terpenes and esters also showed high rOAV values, with (E)-β-damascenone (47,246) and methyl anthranilate (8584) as representative compounds, contributing significantly to the floral and fruity notes of jasmine tea. In contrast, aldehydes such as 2,4-undecadienal decreased significantly after scenting, reflecting the transfer of jasmine-derived aroma components to the tea base. Benzenemethanethiol, a sulfur-containing compound, showed exceptionally high activity in jasmine flowers, indicating that it may play a synergistic role in the overall aroma profile despite not being a typical floral component.

Based on the global screening above, OPLS-DA models were further employed to identify differential compounds between sample groups, with particular focus on those differential compounds exhibiting high rOAV values, to elucidate the transfer and transformation patterns of key aroma compounds during the scenting process. Complete rOAV data are provided in Supplementary Table S2.

### 3.3. Transfer Analysis of Key Aroma Compounds

#### Identification of Differential and Key Aroma-Active Compounds

**Figure 4:**
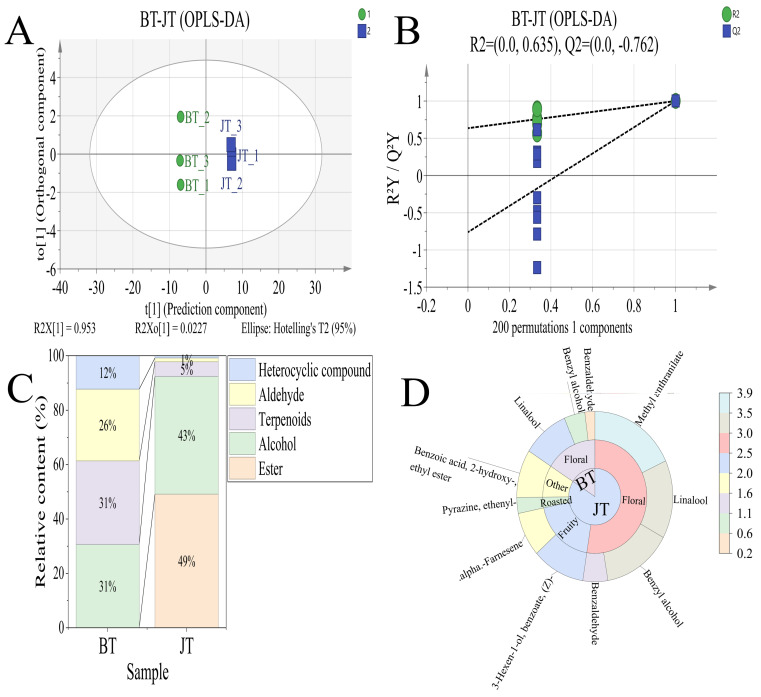
Screening results of aroma compounds in tea base (BT) and jasmine tea (JT). (**A**) Orthogonal Partial Least Squares Discriminant Analysis (OPLS-DA) score plot; (**B**) Permutation test plot (200 iterations); (**C**) Stacked bar chart of differential volatile compounds by chemical class; (**D**) Sunburst chart of key aroma compounds.

**Figure 5:**
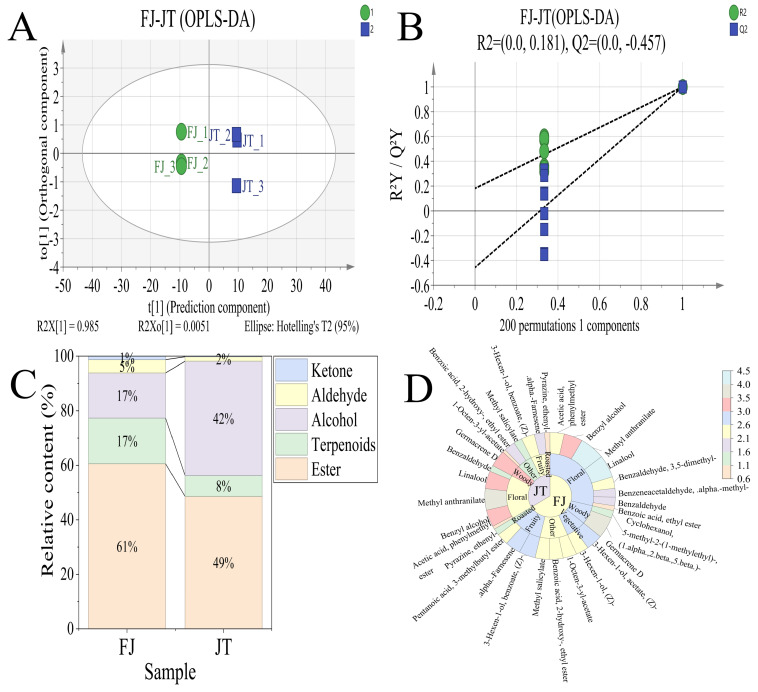
Screening results of aroma compounds in jasmine flowers (FJ) and jasmine tea (JT). (**A**) Orthogonal Partial Least Squares Discriminant Analysis (OPLS-DA) score plot; (**B**) Permutation test plot (200 iterations); (**C**) Stacked bar chart of differential volatile compounds by chemical class; (**D**) Sunburst chart of key aroma compounds.

**Figure 6:**
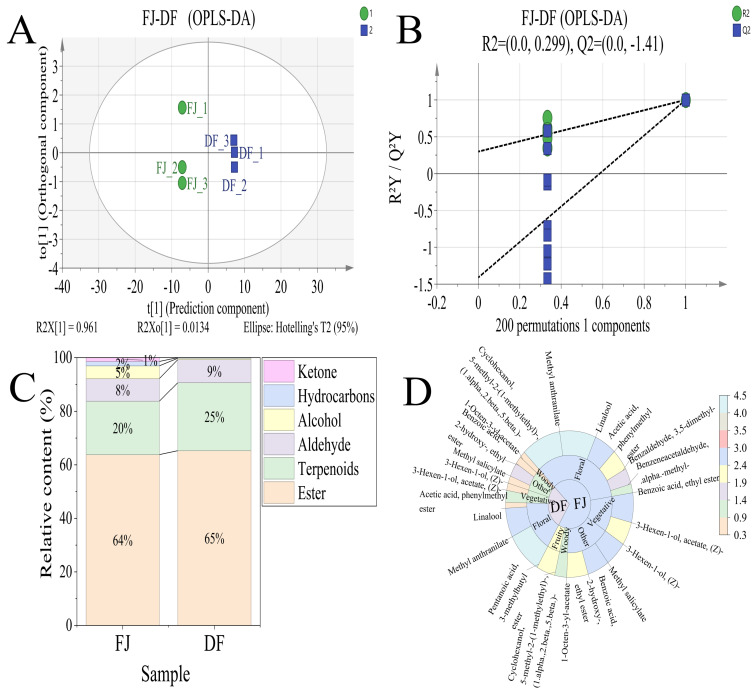
Screening results of aroma compounds in jasmine flowers (FJ) and dried jasmine flowers (DF). (**A**) Orthogonal Partial Least Squares Discriminant Analysis (OPLS-DA) score plot; (**B**) Permutation test plot (200 iterations); (**C**) Stacked bar chart of differential volatile compounds by chemical class; (**D**) Sunburst chart of key aroma compounds.

To trace the migration patterns of aroma compounds during the scenting process, orthogonal partial least squares-discriminant analysis (OPLS-DA) was employed to perform pairwise comparisons of three key sample groups: BT vs. JT, JT vs. FJ, and FJ vs. DF. The OPLS-DA score plots for each comparison group are shown in Figure 4A, Figure 5A and Figure 6A, demonstrating clear intergroup differentiation across all models. The BT sample showed tighter clustering, whereas the FJ, JT, and DF samples were more dispersed. Model quality parameters indicated R^2^Y and Q^2^ values exceeding 0.9. These results suggest robust explanatory and predictive capabilities. Permutation tests (200 permutations) further supported model robustness: as shown in Figure 4B, Figure 5B and Figure 6B, all randomized Q^2^ values were lower than the original Q^2^, and the Q^2^ regression line intercepts were negative, suggesting that the observed group separation is statistically significant and unlikely to be due to overfitting.

To elucidate the overall contribution patterns of different chemical classes to intergroup differences, differential volatiles were categorized by chemical class, and stacked plots were generated based on their relative abundances (Figure 4C, Figure 5C and Figure 6C). Each stacked bar in the figure represents a comparison (e.g., tea base vs. jasmine tea). The bar height indicates the total relative content of differential volatiles in that group, while the height of the color blocks reflects the relative proportions of major substance categories such as esters, alcohols, terpenes, aldehydes, and ketones. This approach reveals, at the chemical class level, the compositional characteristics and structural differences of the differential volatile compounds between groups.

To further focus on key components contributing to actual flavor, we screened for key aroma-active compounds with rOAV ≥ 1 [37] (specific substances listed in Supplementary Table S3) and plotted their rOAV sunburst diagrams (Figure 4D, Figure 5D and Figure 6D). This visualizes the distribution of these key compounds across different samples and their relative contribution strengths, thereby identifying core substances driving sensory differences at the monomer level.

In the comparison between BT and JT, eight differentially volatile compounds were identified. To elucidate the main chemical classes driving the aroma differences between the two groups, a stacked bar chart was constructed based on the relative content of these compounds (Figure 4C).

Analysis revealed fundamental differences in the class distribution of differentially volatile compounds between the two groups. Compared to JT, BT exhibited higher relative proportions of alcohols, terpenes, and aldehydes. Conversely, JT was distinguished by dominant contributions from esters and alcohols. Notably, esters emerged as the most significant contributing class distinguishing jasmine tea from the tea base, accounting for as high as 49% of the relative content, whereas this class did not show a notable contribution in the differential composition of the tea base. Concurrently, terpenes and aldehydes—the primary sources of differentiation in the tea base—dramatically decreased in importance within the differential composition of jasmine tea.

These results demonstrate that the scenting process significantly altered the class distribution pattern of differential volatile compounds: the introduction and accumulation of esters became the most critical factor shaping the distinctive aroma characteristics of jasmine tea, while the attenuation of differences in endogenous terpenes and aldehydes jointly contributed to the directed transformation of the aroma profile.

The top five differential compounds ranked by VIP value were methyl anthranilate, linalool, benzyl alcohol, cis-3-hexenyl benzoate, and α-farnesene—all documented as key jasmine aroma components in the literature [28,38,39]. Among these, methyl anthranilate—identified as the ester class representative in the global rOAV screening (Section 3.2.2, Table 1)—showed the most pronounced change: it was not detected in BT, whereas after scenting, its rOAV in JT reached as high as 8584.39. This de novo introduction established it as a key component shaping the characteristic floral aroma of jasmine tea (Figure 4D) [40]. In contrast, the rOAV values of compounds characteristic of the tea’s inherent flavor, such as ethylpyrazine [41], remained stable before and after scenting. This indicates that while introducing floral aromas, the scenting process effectively preserved the foundational flavor skeleton of the tea, thereby supporting the layered and harmonious character of the final product’s flavor.

In the comparison between JT and FJ, a total of 34 differentially volatile compounds were identified. To elucidate the main chemical class sources driving the differences between the groups, a stacked bar chart was constructed based on the relative abundance of these differentially volatile compounds (Figure 5C). The analysis revealed significant distinctions in the class distribution of volatiles contributing to the intergroup differences. Specifically, compared to JT, the differential composition of FJ was characterized by higher proportions of esters and terpenes. In contrast, the differential characteristics of JT compared to FJ were dominated by esters and alcohols. Notably, the relative contribution of alcohols in the differential composition of jasmine tea (42%) was significantly higher than that in jasmine flowers (17%), whereas the relative contribution of esters (49%) was lower than that in jasmine flowers (61%).

This systematic shift in the composition of differential substances reveals that the scenting process is not merely a transfer of jasmine flower aroma. The results suggest that the tea matrix may exert selective adsorption, retention, or transformation effects on jasmine flower volatiles. The selective retention or generation of alcohols in the tea leaves enhanced their importance in the differential characteristics, while the relative decrease in the contribution of esters jointly shaped the unique aroma profile of jasmine tea that distinguishes it from jasmine flowers.

Analysis of key aroma-active compounds further revealed the specific drivers underlying the aforementioned category shifts (Figure 5D). In the differential composition between jasmine tea and jasmine flowers, the significant enhancement of the contribution from alcohols was primarily attributed to linalool and benzyl alcohol. Although the rOAVs of these two compounds in jasmine tea (1700.14 and 1317.70, respectively) remained significantly lower than those in jasmine flowers (12,978.56 and 1946.27), they exhibited a dramatic increase of more than 13-fold and 175-fold, respectively, compared to the tea base, establishing them as key contributors to the enhanced floral aroma of jasmine tea [42]. This selective enrichment effect, driven by the scenting process, rendered alcohols an important differential class distinguishing jasmine tea from the inherent background of jasmine flowers.

In contrast, although esters remained dominant in the differential composition of jasmine tea (49%), their relative contribution was notably lower than that on the jasmine flower side (61%). This may be because the inherently high contribution of esters in fresh flowers was not proportionally replicated in jasmine tea, resulting in a relative decrease in their differential contribution [43].

Furthermore, the marked difference in the magnitude of increase between linalool and benzyl alcohol (13-fold vs. 175-fold) further suggests that the tea matrix may exert differential regulation on various aroma compounds in terms of adsorption affinity, retention efficiency, or transformation rates.

In the comparison between FJ and DF, a total of 22 differential volatile compounds were identified. A stacked bar chart was generated based on the relative class contents of these differential volatile compounds (Figure 6C) to elucidate the chemical class sources of the differences between the groups.

Unlike the previous comparison groups, the overall class distribution of differential volatile compounds between FJ and DF was highly similar. In both groups, esters were the dominant differential contribution category (FJ: 64%, DF: 65%), followed by terpenes (FJ: 20%, DF: 25%). This stability indicates that, after aroma release during scenting and subsequent drying treatment, DF did not undergo a significant shift in the major class contribution pattern of differential volatiles compared to FJ. Esters and terpenes still occupied the dominant and sub-dominant positions, respectively, with their relative contribution proportions remaining essentially unchanged from those in FJ.

However, beneath this apparent stability in class composition, a significant reconstitution of specific components within each class occurred. Examining the specific compounds (Figure 6D), several key aroma components in FJ showed substantial decreases in the DF after undergoing release during scenting and subsequent drying. For example, methyl anthranilate decreased from 33,149.59 to 14,077.05, and linalool decreased from 12,978.56 to 791.81, yet their value remained far higher than the background levels in BT. It is these dramatic changes in the content of key components that render them the core metabolites defining the differences in this group (FJ vs. DF). Particularly noteworthy is that cis-3-hexenyl benzoate still maintained a significant aroma contribution in DF (rOAV: 654.74) [44]. The marked differences in the extent of rOAV reduction among various aroma compounds intuitively reflect their divergent comprehensive retention efficiency and transformation pathways during the aroma release phase of scenting and the subsequent baking processing, which may be closely related to their physicochemical properties, such as volatility, thermal stability, and interaction with the tea matrix.

### 3.4. Correlation Analysis of Physicochemical Properties of Differential Aroma Compounds with Their Transfer Rates and Retention Rates

**Figure 7:**
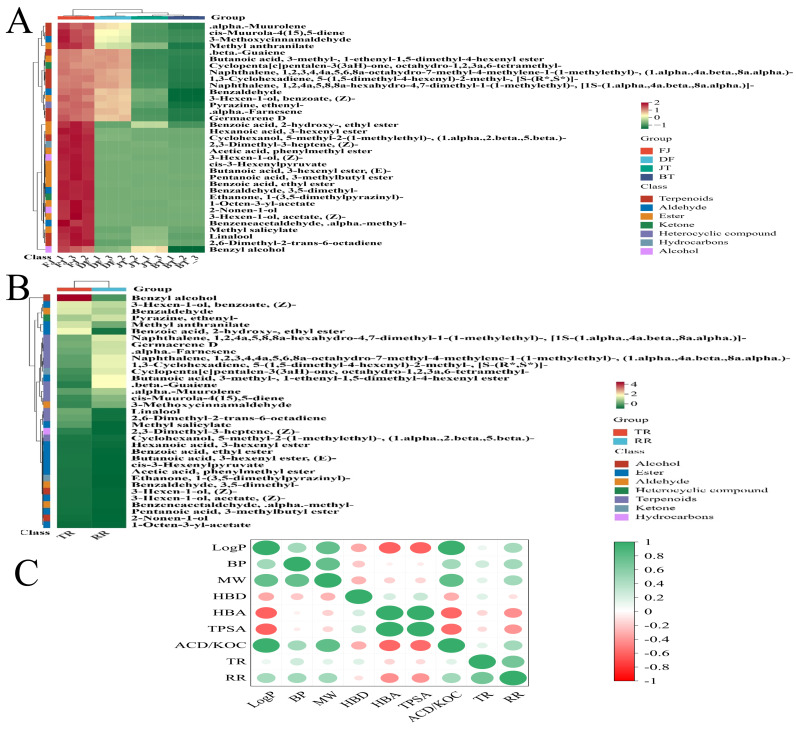
Transfer dynamics of aroma compounds during scenting. (**A**) Heatmap of relative content changes in intergroup differential volatile compounds; (**B**) Heatmap of transfer rates (TRs) from jasmine flowers to tea base and retention rates (RRs) in dried jasmine flowers; (**C**) Correlation analysis between physicochemical properties (boiling point (BP), octanol-water partition coefficient (logP), molecular weight (MW), topological polar surface area (TPSA), hydrogen bond donor count (HBD), hydrogen bond acceptor count (HBA), organic carbon–water partition coefficient (ACD/KOC)) of differential compounds and their TR and RR.

Based on rOAV and content analysis, the characteristic flavor of jasmine tea primarily originates from the introduction of high sensory-contributing compounds from jasmine flowers, such as methyl anthranilate, linalool, and benzyl alcohol. To elucidate their transfer patterns, this study generated a heatmap of relative content variations for differential volatile compounds and calculated both their transfer rates (TRs) from jasmine flowers to the tea base and their retention rates (RRs) in the dried jasmine flowers (Figure 7A,B).

The heatmap (Figure 7A) shows that the relative contents of the differential metabolites generally followed the trend FJ > DF > JT > BT, directly reflecting the systematic redistribution of aroma compounds along the “release-adsorption-residue” pathway. Further analysis of TR and RR (Figure 7B) revealed significant differences in the transfer behaviors of different components, which could be classified into three categories: (1) High-transfer type: represented by characteristic jasmine esters and alcohols, such as benzyl alcohol (TR: 67.32%), cis-3-hexenyl benzoate (TR: 27.73%), and methyl anthranilate (TR: 25.90%). As evident from the above analysis, these compounds are also key active components identified in the rOAV analysis, and their efficient transfer directly constitutes the characteristic aroma base of jasmine tea. (2) High-loss type: some compounds exhibited even lower contents in jasmine tea than in the tea base, resulting in negative transfer rates, such as 1-octen-3-yl acetate (TR: −1.62%) and 2-nonen-1-ol (TR: −0.50%). This may be related to their high volatility or degradation under hot and humid conditions. (3) Trace residue type: compounds such as 3,5-dimethylbenzaldehyde (TR: 0.67%, RR: 0%) and 1-(3,5-dimethylpyrazin-2-yl) ethan-1-one (TR: 0.73%, RR: 0%) showed almost no residue in the dried jasmine flowers, suggesting that they were completely volatilized during the scenting process.

In summary, the transfer pathways of aroma compounds during the scenting process are highly specific, indicating that their transfer efficiency may be jointly regulated by their physicochemical properties and interactions with the tea matrix [45].

To investigate the physicochemical principles underlying the observed differences in transfer behavior, key physicochemical parameters of the differential volatile compounds, including boiling point (BP), octanol-water partition coefficient (logP), molecular weight (MW), topological polar surface area (TPSA), hydrogen bond donor/acceptor counts (HBD/HBA), and organic carbon-water partition coefficient (ACD/KOC), were retrieved from public databases and analyzed for their associations with TR and RR using Spearman’s correlation analysis (Figure 7C).

The results indicated that RR was significantly positively correlated with hydrophobicity (logP, r = 0.46, *p* = 0.007), solid-phase adsorption potential (ACD/KOC, r = 0.47, *p* = 0.006), and molecular size (MW, r = 0.47, *p* = 0.006), while being significantly negatively correlated with polarity (TPSA, r = −0.42; HBA, r = −0.44, *p* < 0.05). This suggests that the retention of aroma in dried flowers may be primarily regulated by a hydrophobic partitioning mechanism: compounds with stronger hydrophobicity, higher adsorption potential, and larger molecular size tend to be more readily retained in the lipid or hydrophobic regions of the dried flower tissue, whereas polar molecules, due to their higher hydrophilicity, are more prone to loss during water transfer.

In contrast, TR from flowers to tea exhibited a significant positive association with RR (r = 0.69, *p <* 0.001) but showed no significant monotonic correlations with any single physicochemical parameter, such as logP, polarity, or molecular weight (|r| < 0.26, *p* > 0.15). This suggests that the entire process of aroma release from flowers to adsorption by tea leaves constitutes a multi-step, complex mass transfer system, whose overall efficiency may be jointly determined by volatility kinetics, diffusion efficiency within the porous tea matrix, and specific interactions with tea components, rather than being linearly dominated by a single thermodynamic parameter [46,47].

Furthermore, the physicochemical parameters exhibited high synergy: logP, ACD/KOC, molecular weight, and boiling point were strongly positively correlated (r > 0.76, *p* < 0.05), forming a hydrophobicity-partitioning potential parameter cluster that jointly and positively regulated the RR. The number of HBA and TPSA were nearly collinear (r = 0.99, *p* < 0.05), constituting a polarity-hydrophilic parameter cluster that jointly and negatively influenced the RR. The number of hydrogen bond donors (HBDs) showed no significant correlation with any transfer indicators, indicating that it is not a key regulatory variable in this system.

## 4. Discussion

### 4.1. Key Aroma-Active Compounds and Cross-Methodological Consistency

To identify the key contributors to jasmine tea aroma, we first performed OPLS-DA to identify differential compounds between sample groups and then combined these results with rOAV analysis to assess their aroma activity. The top five VIP compounds—methyl anthranilate, linalool, benzyl alcohol, cis-3-hexenyl benzoate, and α-farnesene—were identified as key differential compounds, all exhibiting high rOAV values in jasmine tea.

These key compounds align closely with recent advances in jasmine tea aroma research. Qi et al. [11] employed GC-O to identify key aroma compounds in jasmine tea, including methyl anthranilate and linalool, both of which correspond to the top VIP compounds in our study. Their GC-O analysis confirmed that these compounds are aroma active, consistent with the high rOAV values observed in our work. This cross-methodological agreement between our rOAV-based approach and their GC-O analysis provides strong validation for the importance of these compounds, despite the absence of direct GC-O measurements in our study.

Similarly, Li et al. [12] constructed a comprehensive aroma wheel for jasmine tea based on volatile compound profiling during multiple scenting processes. Their aroma wheel was dominated by floral, fruity, and green notes, with key compound classes including green leaf volatiles, benzyl derivatives, and terpenes. These compound classes directly correspond to the key compounds identified in our study. The consistency between our findings and their aroma wheel classification reinforces the robustness of these compounds as core markers of jasmine tea aroma.

### 4.2. Transfer and Retention Mechanisms of Aroma Compounds

The absence of significant monotonic correlations between TR and individual physicochemical parameters such as logP, polarity, or molecular weight suggests that the overall transfer process may not be governed by a single thermodynamic parameter but may involve a multi-step mass transfer system. In this system, volatility kinetics, diffusion within the porous tea matrix, and specific interactions with tea components may collectively influence transfer efficiency [46,47].

In contrast, the retention of aroma compounds in spent flowers exhibited clear physicochemical dependence. RR was positively correlated with hydrophobicity-related parameters and negatively correlated with polarity-related parameters, with these hydrophobicity-related parameters being strongly intercorrelated. This suggests that retention may be primarily driven by hydrophobic partitioning effects, a pattern consistent with reports in food nanoencapsulation systems, where hydrophobic flavor compounds tend to be retained in the hydrophobic regions of the carrier matrix [48]. By analogy, during the dehydration of jasmine flowers, the hydrophobic microenvironments within their cells—such as lipid membranes and waxy layers—may exert a similar entrapment effect on hydrophobic aroma compounds, which may help explain their relatively high residue in dried flowers [49].

Collectively, the contrasting correlation patterns between TR and RR suggest that the transfer and retention of aroma compounds during scenting may be governed by distinct mechanisms: transfer may involve a multi-step mass transfer process, whereas retention may be driven primarily by hydrophobic partitioning effects.

### 4.3. Methodological Considerations and Future Perspectives

Several methodological considerations should be noted. First, the OPLS-DA models were constructed using three technical replicates from a single production batch, which limits generalizability. Rigorous internal validation confirmed model robustness, and the consistency of key compounds with independent studies [11,12] provides indirect external validation. Future studies incorporating multiple independent batches are needed to confirm the robustness of these markers.

Second, the absence of GC-O analysis means that actual aroma activity was calculated from rOAV rather than directly measured. However, the strong agreement between our rOAV-based findings and GC-O results from independent studies [11] supports the reliability of this approach. Future studies incorporating GC-O analysis would further validate the aroma activity of the identified compounds.

Third, the correlation analysis between physicochemical parameters and transfer/retention rates, while informative, is based on a limited set of compounds. Expanding the dataset with a broader range of compounds would help establish more generalizable relationships.

## 5. Conclusions

In conclusion, this study reveals that the transfer and retention of aroma compounds during jasmine tea scenting exhibit different patterns. Transfer rates of aroma compounds showed no significant monotonic correlation with single physicochemical parameters, implying a multi-step mass transfer process. Retention rates in spent jasmine flowers showed a positive association with hydrophobicity-related parameters and a negative relationship with polarity-related parameters, implying that hydrophobic partitioning effects may play a predominant role. Five key aroma compounds were identified as major contributors to jasmine tea aroma, namely methyl anthranilate, linalool, benzyl alcohol, cis-3-hexenyl benzoate, and α-farnesene. These findings provide a theoretical basis for optimizing scenting parameters to improve aroma utilization efficiency. Future studies with multi-batch and GC-O validation are warranted to further generalize these results.

## Figures and Tables

**Table 1:** Representative aroma-active compounds of each chemical class.

Class	Representative Compound	rOAV (FJ)	rOAV (JT)	Odor Description	Origin
Terpenes	(E)-β-Damascenone	969,888	47,246	Fruity, floral	FJ
Esters	Methyl anthranilate	33,150	8584	Grape, fruity	FJ
Aldehydes	2,4-Undecadienal	47,486	3200	Green	FJ
Ketones	Dihydro-2-methyl-3(2H)-furanone	412,082	570,251	Sweet	BT
Alcohols	Benzenemethanethiol	67,669	12,532	Onion, garlic	FJ
Heterocyclics	3-Methylindole	34	24	Animal-like	FJ
Phenols	Eugenol	1789	296	Floral	FJ
Sulfur compounds	Diallyl sulfide	14	17	Sulfurous	BT/FJ
Acids	4-Methylpentanoic acid	5	1	Cheesy	FJ

Abbreviations: BT, tea base; FJ, fresh jasmine flowers; JT, jasmine tea; rOAV, relative odor activity value. Complete rOAV data, odor thresholds, and CAS numbers are provided in Supplementary Table S2.

**Table 2:** Top five terpenes in jasmine tea (JT) ranked by relative odor activity value (rOAV).

Rank	Compound	rOAV (JT)	Odor Description
1	(E)-β-Damascenone	47,246	Fruity, floral
2	Linalool	1700	Floral, green
3	Germacrene D	1117	Woody
4	(−)-Caryyl acetate	673	Green
5	α-Farnesene	67	Citrus, herbal

Complete rOAV data and odor thresholds are provided in Supplementary Table S2.

## Data Availability

The raw data supporting the findings of this study are provided within the article and its Supplementary Materials. Physicochemical parameters of volatile compounds (e.g., boiling point, logP, molecular weight, TPSA, HBD, HBA, ACD/KOC) were retrieved from public chemical databases, specifically ChemSpider (https://www.chemspider.com/) and PubChem (https://pubchem.ncbi.nlm.nih.gov/). Any additional inquiries concerning the original data may be addressed to the corresponding authors.

## Supplementary Materials

The following supporting information can be downloaded at https://www.mdpi.com/article/10.3390/foods15081403/s1. Table S1: Summary of Changes in Different Aroma Component Concentrations During Jasmine Tea Scenting Process; Table S2: Relative odor activity values (rOAV) of aroma compounds during the scenting process of jasmine tea; Table S3: Summary of rOAV Values for Key Aroma Compounds During Jasmine Tea Scenting Process.
